# Transient receptor potential cation channel 6 contributes to kidney injury induced by diabetes and hypertension

**DOI:** 10.1152/ajprenal.00296.2021

**Published:** 2021-12-06

**Authors:** Zhen Wang, Yiling Fu, Jussara M. do Carmo, Alexandre A. da Silva, Xuan Li, Alan Mouton, Ana Carolina M. Omoto, Jaylan Sears, John E. Hall

**Affiliations:** ^1^Department of Physiology and Biophysics, University of Mississippi Medical Center, Jackson, Mississippi; ^2^Mississippi Center for Obesity Research, University of Mississippi Medical Center, Jackson, Mississippi

**Keywords:** albumin excretion, apoptosis, glomerular filtration, nephropathy, type 1 diabetes

## Abstract

Diabetes mellitus (DM) and hypertension (HTN) are major risk factors for chronic kidney injury, together accounting for >70% of end-stage renal disease. In this study, we assessed whether DM and HTN interact synergistically to promote kidney dysfunction and whether transient receptor potential cation channel 6 (TRPC6) contributes to this synergism. In wild-type (WT; B6/129s background) and TRPC6 knockout (KO) mice, DM was induced by streptozotocin injection to increase fasting glucose levels to 250–350 mg/dL. HTN was induced by aorta constriction (AC) between the renal arteries. AC increased blood pressure (BP) by ∼25 mmHg in the right kidney (above AC), whereas BP in the left kidney (below AC) returned to near normal after 8 wk, with both kidneys exposed to the same levels of blood glucose, circulating hormones, and neural influences. Kidneys of WT mice exposed to DM or HTN alone had only mild glomerular injury and urinary albumin excretion. In contrast, WT kidneys exposed to DM plus HTN (WT-DM + AC mice) for 8 wk had much greater increases in albumin excretion and histological injury. Marked increased apoptosis was also observed in the right kidneys of WT-DM + AC mice. In contrast, in TRPC6 KO mice with DM + AC, right kidneys exposed to the same levels of high BP and high glucose had lower albumin excretion and less glomerular damage and apoptotic cell injury compared with right kidneys of WT-DM + AC mice. Our results suggest that TRPC6 may contribute to the interaction of DM and HTN to promote kidney dysfunction and apoptotic cell injury.

**NEW & NOTEWORTHY** A major new finding of this study is that the combination of moderate diabetes and hypertension promoted marked renal dysfunction, albuminuria, and apoptotic cell injury, and that these effects were greatly ameliorated by transient receptor potential cation channel 6 deficiency. These results suggest that transient receptor potential cation channel 6 may play an important role in contributing to the interaction of diabetes and hypertension to promote kidney injury.

## Graphical abstract

**Figure:**
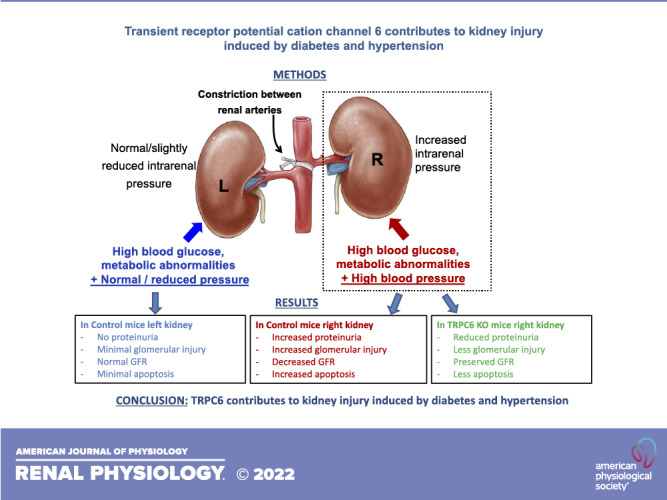

## INTRODUCTION

The prevalence of diabetes mellitus (DM) has increased dramatically in the United States during the past several decades. Hypertension (HTN) occurs twice as frequently in patients with DM as compared with the general population, with a prevalence of ∼25% in young patients with type 1 DM and 50–85% in older patients with type 2 DM (1–3). The coexistence of DM and HTN, especially when they are not adequately controlled, substantially increases the risk for the onset and progression of chronic kidney disease. Although current therapeutic options may slow the progression of diabetic-hypertensive nephropathy, many of these patients ultimately progress to end-stage renal disease (4–6). Therefore, there is an urgent need to identify the mechanisms responsible for diabetic-hypertensive nephropathy and to develop new and more effective therapeutic targets.

Transient receptor potential (TRP) cation channel 6 (TRPC6) is a member of the TRP superfamily of nonselective cation channels expressed widely in kidney cells, including mesangial cells, the endothelium, and podocytes within the glomerulus (7). TRPC6 activation leads to Ca^2+^ influx that can be receptor mediated or by mechanically and osmotically induced membrane stretch (8). A growing interest in the potential role of TRPC6 channels in kidney injury emerged after the discovery that gain-of-function mutations in TRPC6 cause focal segmental glomerulosclerosis (9). Increased expression of TRPC6 was also found in podocytes of patients and animals with diabetic proteinuric kidney disease, consistent with the possibility that excessive activation of TRPC6 and subsequent Ca^2+^ influx may be a common pathway for glomerular dysfunction (7, 10).

In the present study, we determined *1*) whether hyperglycemia in type 1 DM and moderate HTN synergistically promote kidney injury and *2*) whether TRPC6 plays a role in contributing to kidney injury induced by the combination of DM and HTN. We developed a mouse model that combined type 1 DM and HTN induced by aorta constriction (AC) between the renal arteries in TRPC6 knockout (KO) and wild-type (WT) control mice. Using type 1 DM rather than type 2 DM model in this study simplifies the contributing factors for renal injury to hyperglycemia and AC-induced high blood pressure (BP) and avoids complicating effects of obesity often associated with type 2 DM models. An important feature of this model is that both kidneys are exposed to the same levels of hyperglycemia, circulating hormones, and neural influences, but the left kidney below the AC has normal to slightly reduced BP, whereas the right kidney above the AC is exposed to elevated BP. Therefore, we could compare left and right renal structure and function in the same animal to determine the direct impact of DM or increased BP alone in promoting kidney injury as well as the combined effects of DM and HTN.

Our results indicate that in WT mice, kidneys exposed to hyperglycemia plus HTN had greater glomerular injury and urinary albumin excretion than kidneys exposed to only hyperglycemia or HTN. TRPC6 KO mice, however, were protected from the synergistic impact of DM and HTN to promote albuminuria, glomerular injury, and apoptosis in kidneys exposed to similar levels of hyperglycemia and elevated BP as in WT mice. These results suggest that TRPC6 may play an important role in contributing to the interaction of DM and HTN to promote kidney injury.

## MATERIALS AND METHODS

### Animals

The experimental procedures described in this study followed the National Institutes of Health *Guide for the Care and Use of Laboratory Animals* and were approved by the Institutional Animal Care and Use Committee of the University of Mississippi Medical Center. TRPC6 KO mice (B6;129S-Trpc6tm1Lbi/Mmjax) and WT B6/129s control mice from Jackson Laboratories were bred in our animal facility. The phenotype of TRPC6 KO mice has been described in a previous study (11) and confirmed by genotyping in our laboratory. Previous studies also showed that angiotensin II evoked intracellular Ca^2+^ transients in podocytes isolated from WT mice, but these transients were markedly blunted in TRPC6 KO mice (12, 13). The B6/129s hybrid mice used in these experiments were the offspring of more than four generations of an F1 × F1 crossing between C57BL/6J females (B6) and 129S1/SvImJ males (129S) and served as controls for TRPC6 KO mice that were generated with 129-derived embryonic stem cells and maintained on a mixed B6/129 background.

Mice had free access to standard chow (Harlan Laboratories) and were housed in individual cages maintained at 23 ± 2°C with a 12:12-h light-dark cycle. Mice were randomly divided into the following six groups in this study (Table 1): *groups 1* and *2*, WT and TRPC6 KO mice with type 1 DM (WT-DM and TRPC6 KO-DM), respectively; *groups 3* and *4*, nondiabetic WT and TRPC6 KO mice with AC surgery-induced HTN (WT-AC and TRPC6 KO-AC), respectively; and *groups 5* and *6*, WT and TRPC6 KO mice with both type 1 DM and HTN (WT-DM + AC and TRPC6 KO-DM + AC), respectively.

**Table 1.** Experimental groups of WT and TRPC6 KO mice

Group	Animal	Procedure	Phenotype
DM only	TRPC6 KO-DM	STZ injection and then sham surgery	Both kidneys exposed to DM, no HTN
	WT-DM		
HTN only	TRPC6 KO-AC	Saline injection and then AC surgery	Right kidney exposed to HTN and left kidney exposed to normal/slightly reduced blood pressure
	WT-AC		
DM + HTN	TRPC6 KO-DM + AC	STZ injection and then AC surgery	Right kidney exposed to DM + HTN and left kidney exposed to DM with normal/slightly reduced blood pressure
	WT-DM + AC		

AC, aortic constriction; DM, diabetes mellitus; HTN, hypertension; KO, knockout; STZ, streptozotocin; TRPC6, transient receptor potential cation channel 6; WT, wild-type.

### Experimental Protocol

**Figure 1.**
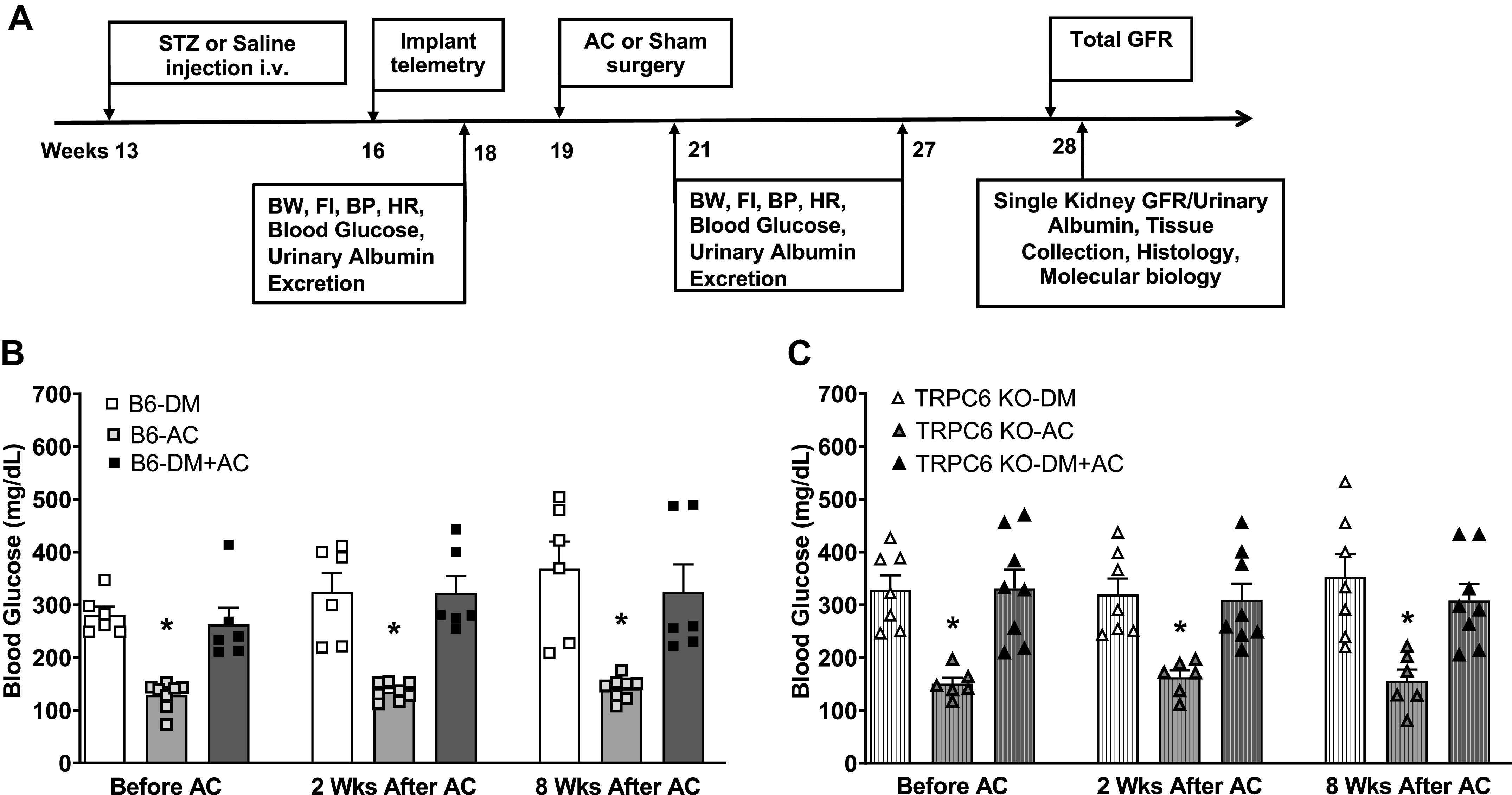
Blood glucose concentrations in different groups of male wild-type (WT) and transient receptor potential cation channel 6 (TRPC6) knockout (KO) mice before and after aortic constriction (AC) or sham surgery. *A*: study protocol. *B*: fasting blood glucose concentrations in diabetes mellitus (DM), AC, and DM + AC groups of WT mice before AC or sham surgery and at 2 and 8 wk after the surgery. *C*: fasting blood glucose concentrations in DM, AC, and DM + AC groups of TRPC6 KO mice before AC or sham surgery and at 2 and 8 wk after the surgery. *n* = 6–8 per group. *P < 0.05 when comparing AC vs. DM or DM + AC mice by one-way ANOVA and Tukey’s test. BP, blood pressure; B6, C57BL/6J females; BW, body weight; FI, food intake; GFR, glomerular filtration rate; HR, heart rate; STZ, streptozotocin.

Type 1 DM was induced in mice at 13 wk of age with an injection of streptozotocin (STZ; Sigma-Aldrich) at 125 mg/kg through the penile dorsal vein to induce insulin-deficient type 1 DM. We used only male mice in our study to produce stable and reproducible hyperglycemia with a moderate dose of STZ to avoid high-dose STZ-related renal toxicity, which was difficult to reproduce in female mice. Mice with blood glucose levels stabilized around 300 ± 50 mg/dL were used for the next step. At 16 wk of age, BP telemetry transmitters were implanted in the mice, followed by 7 days of recovery. Mean arterial pressure (MAP), heart rate (HR), food intake, urine output, urinary albumin excretion, and blood glucose were measured 24 h/day for 5 consecutive days as baseline control period values. At 19 wk of age, AC or sham surgeries were performed, followed by another 7 days of recovery. MAP, HR, food intake, urine output, urinary albumin excretion, and blood glucose were measured for 3 consecutive days at 2 and 8 wk after the AC or sham surgery. At 28 wk of age, total glomerular filtration rate (GFR) was measured, followed by measurements of separate left and right kidney GFR and urinary albumin excretion. Both kidneys were harvested at the end of the in vivo experiment and stored for morphology, Western blot analysis, and immunohistochemistry experiments (Fig. 1*A*).

### Implantation of the Telemetry Transmitter

At 16 wk old, WT and TRPC6 KO mice were anesthetized with 2% isoflurane and a telemetry probe (TA11PA-C10, Data Science) was implanted in the left carotid artery and advanced into the aorta. Seven to ten days after recovery from the surgery, MAP and HR were measured by telemetry 24 h/day for 5 consecutive days using computerized methods for data collection as previously described (10, 13). Daily MAP and HR were obtained from the average of 12:12-h light-dark recording using a sampling rate of 1 Hz and a duration of 30 s every 10 min.

### AC Surgery to Induce HTN in Mice

For the AC surgery, mice were placed in the right lateral position and body temperature was maintained at 37°C with a heating pad. A ∼2- to 3-cm skin incision was made in the left lateral flank 1 cm below the rib cage. After the left kidney had been exposed and following the left renal artery to the aorta, a 5-0 silk suture was wrapped around the aorta between the left and right renal arteries. A cut sterile 29-gauge needle (0.5 cm long) was placed next to the aorta, and a surgeon’s knot was snugly tied around the needle and aorta to produce a consistent and precise degree of constriction. The needle was then removed from the suture loop and the muscles and skin were closed. The needle between two renal arteries was carefully gauged to cause a mild reduction in BP in the left kidney rather than induce major ischemia below the constriction. To determine the BP gradient above and below the constriction, a femoral artery catheterization was performed under anesthesia on the last day of the experiment. After cannulation, the catheter was connected to PowerLab data-acquisition system (ADInstruments) to record BP. Simultaneously, BP was measured from the telemetry catheter implanted in the common carotid artery and advanced into the aortic arch above the AC. The pressure gradients across the AC averaged 20 ± 4 mmHg.

### Total GFR Measurements in Conscious Mice With 24-h FITC-Inulin Infusion

Eight weeks after the AC or sham surgery, WT and TRPC6 KO mice were implanted with venous catheters in the jugular vein for infusion of FITC-inulin (Sigma-Aldrich). The venous catheter was tunneled subcutaneously, exteriorized between the scapulae, and passed through a spring connected to a mouse swivel (Instech) mounted on the top of the plastic metabolic cage. The venous catheter was connected through a sterile filter to a syringe pump for continuous saline infusions. After 5 days of recovery, 0.25% FITC-inulin dissolved in saline was infused at a rate of 0.135 mL/h for 24 h; 100 µL of blood was collected through the tail vein 15 min before the end of the infusion. Total GFR was calculated based on the inulin clearance by measuring the volume of FITC-inulin infused during 24 h and the concentrations of FITC-inulin in the blood and syringe using a fluorescence plate reader with the wavelength of excitation at 490 nm and emission at 525 nm. The formula for GFR in conscious animals is as follows: Inulin clearance (GFR) = FITC-inulin infusion rate/steady-state blood FITC-inulin concentration (14–16).

### Separate Kidney GFR and Urine Collection

Mice were anesthetized with 1% Inactin (Sigma), the jugular vein catheter was used to allow intravenous infusion, and a femoral artery catheter was used for blood collection. FITC-inulin (0.1%) was infused intravenously at 0.8 mL/h for 90 min, and urine samples from each kidney were collected during the last 60 min. To collect urine from the right kidney, the right ureter was exposed and a tapered catheter (RenaPulse RT 040, Braintree Scientific) was inserted into the lumen of the ureter. A flanged PE-40 size catheter was inserted into the bladder to collect urine from the left kidney as all urine from the right kidney was collected via the ureter catheter. Blood samples (100 µL) were collected at 60 and 90 min of the infusion period, and GFR from each kidney was calculated from the average rate of inulin clearance. The formula for the split kidney GFR is as follows: Inulin clearance (GFR) = urinary FITC-inulin excretion rate/steady-state blood FITC-inulin concentration. GFRs from left and right kidneys were normalized to kidney weight, respectively.

### Blood and Urine Biochemistry Measurements

Fasting blood glucose levels were measured using glucose strips (ReliOn Prime Blood Glucose Test Strips). Fasting plasma insulin and leptin concentrations were measured with ELISA (R&D Systems and Crystal Chem, respectively). Urine albumin levels were determined with ELISA (Crystal Chem) from urine collected for 72 h in mice placed in metabolic cages or urine collected during the separate kidney GFR measurements.

### Western Blot Analysis for Measuring Apoptosis

Proteins of the renal cortex of the left and right kidneys were isolated after homogenization in RIPA lysis buffer. Rabbit anticleaved caspase-3 antibody (No. 9661, Cell Signaling, dilution: 1:1,000) was added, and samples were incubated at 4°C overnight. The specificity of this cleaved caspase-3 (Asp^175^) antibody has been validated by Cell Signaling; it detects endogenous levels of the large fragment (17/19 kDa) of activated caspase-3 resulting from cleavage adjacent to Asp^175^ and does not recognize full-length caspase-3 or other cleaved caspases. Donkey anti-rabbit secondary antibody (IRDye 680RD, LI-COR, dilution: 1:10,000, incubation at room temperature for 1 h) was used in Western blots. Images were acquired with the Odyssey Imaging System (LI-COR Biosciences). Expression levels of cleaved caspase-3 were normalized to expression levels of β-actin (No. 4970, Cell Signaling).

### Renal Histology

Paraffin-embedded sections (5 µm) were prepared from kidneys fixed in 10% phosphate-buffered formalin. Periodic acid-Schiff stain was used for analyses of renal morphology changes. Sections were scored in a blinded, semiquantitative manner using an established scoring scale. For each animal, at least 10 high-power (×400) fields were examined. Percentages of glomeruli that displayed glomerular capillary wall thickening, mesangial expansion, nodular sclerosis, and global glomerulosclerosis were scored as follows: 0 = none, 1 = <25%, 2 = 25–50%, 3 = 50–75%, and 4 = >75%.

### Cleaved Caspase-3 Immunohistochemistry Staining

Left and right kidneys from WT and TRPC6 KO mice were harvested, fixed in 10% formalin for 24 h, embedded in paraffin, and then cut (5 µm) for cleaved caspase-3 staining. Sections were rehydrated, and antigens were unmasked in 10 mM of sodium citrate (pH 6.0) heated at 95°C for 30 min. Serum-free protein blocker (Vector Laboratories) was added, and sections were then incubated with anticleaved caspase-3 antibodies (No. 9661, Cell Signaling, dilution: 1:200) overnight at 4°C in a humid chamber. After being rinsed with PBS, sections were incubated with secondary antibody (1:400) and then subjected to diaminobenzidine staining by a Vector ABC-HRP Kit.

### Statistical Analysis

The number of animals used was based on our previous studies and power analysis to predict a 20% difference among groups. Data are expressed as means ± SE. A *P* value of <0.05 indicates a significant difference. Significant differences between two groups were determined by Student’s *t* test. Significant differences between multiple groups were determined by one-way ANOVA followed by a Tukey’s post hoc test. Significant differences between multiple groups at different time points were determined by two-way ANOVA followed by Tukey’s or Bonferroni’s multiple-comparison tests. Histological scoring in kidneys was assessed by a trained researcher in histological analyses who was blinded to the experimental protocols to avoid bias, and the results were assessed using a nonparametric Kruskal*–*Wallis test followed by a Dunn’s multiple-comparison test. Individual statistical analyses are described in the figures.

## RESULTS

### Metabolic Characteristics of WT and TRPC6 KO Mice With DM, HTN, and DM + HTN

The time line for the animal experiments is shown in Fig. 1*A*. Briefly, type 1 DM was induced in mice at 13 wk of age with STZ injection. After baseline measurements at 19 wk of age, AC or sham surgeries were performed. MAP, HR, food intake, urine output, urinary albumin excretion, and blood glucose were measured at 2 and 8 wk after the AC or sham surgery. At 28 wk of age, GFR was measured, followed by measurements of separate left and right kidney GFR and urinary albumin excretion. Both kidneys were harvested at the end of the in vivo experiment for morphology and immunohistochemistry experiments.

The DM phenotype after STZ injection was confirmed by significantly increased blood glucose levels in WT and TRPC6 KO mice. As shown in Fig. 1*B*, blood glucose levels in WT mice averaged 340 ± 80 mg/dL in the DM group and 302 ± 45 mg/dL in the DM + AC group at the baseline compared with 132 ± 5 mg/dL in saline-injected mice. Blood glucose levels were maintained at similar levels when measured again at 2 and 8 wk after the AC or sham surgery. In TRPC6 KO mice, average blood glucose levels were 303 ± 35 mg/dL in the DM group and 312 ± 33 mg/dL in the DM + AC group at the baseline compared with 151 ± 6 mg/dL in saline-injected mice (Fig. 1*C*). Blood glucose levels in each group of TRPC6 KO mice at 2 and 8 wk after the AC or sham surgery were maintained at levels similar to those observed in WT mice at the same time points.

We also measured body weight and food intake in different groups of WT and TRPC6 KO mice (Table 2) before AC or sham surgery as the baseline and at 2 and 8 wk after surgery. The results showed that in WT mice, the DM, AC, and DM + AC groups had a similar body weight at the baseline. At 8 wk after surgery, WT-DM + AC mice had a slightly lower body weight than WT-AC mice. As expected, diabetic mice (WT-DM and WT-DM + AC) had significantly greater food intake than WT-AC mice. In TRPC6 KO mice, the diabetic groups (DM and DM + AC) had significantly lower body weight and higher food intake than nondiabetic mice at the baseline.

**Table 2.** Body weight and food intake in different groups of male WT and TRPC6 KO mice before and after AC or sham surgery

	WT-DM	WT-AC	WT-DM + AC	TRPC6 KO-DM	TRPC6 KO-AC	TRPC6 KO-DM + AC
Body weight, g						
Before AC	33.1 ± 1.9	33.9 ± 0.9	31.2 ± 1.2	32.9 ± 1.3	36.5 ± 1.6*	32.7 ± 1.1
2 wk after AC or sham	33.8 ± 2.0	34.5 ± 1.1	32.5 ± 1.3	34.2 ± 1.1	37.2 ± 1.7*	32.6 ± 1.1
8 wk after AC or sham	34.6 ± 2.1	38.3 ± 0.4*	33.7 ± 1.3	35.7 ± 2.4	38.4 ± 1.9*	34.5 ± 1.2
Food intake, g						
Before AC	4.4 ± 0.4	3.5 ± 0.1*	4.1 ± 0.3	4.3 ± 0.1	3.5 ± 0.3*	4.8 ± 0.4
2 wk after AC or sham	4.7 ± 0.3	3.4 ± 0.2*	4.2 ± 0.3	4.8 ± 0.1	3.4 ± 0.2*	4.8 ± 0.3
8 wk after AC or sham	4.2 ± 0.4	3.3 ± 0.1*	4.1 ± 0.3	4.5 ± 0.1	3.7 ± 0.1*	4.3 ± 0.2

Values are means ± SE; *n* = 6–8 per group. AC, aortic constriction; DM, diabetes mellitus; KO, knockout; TRPC6, transient receptor potential cation channel 6; WT, wild-type. *P < 0.05 when comparing AC vs. DM + AC mice by one-way ANOVA and Tukey’s test.

**Figure 2.**
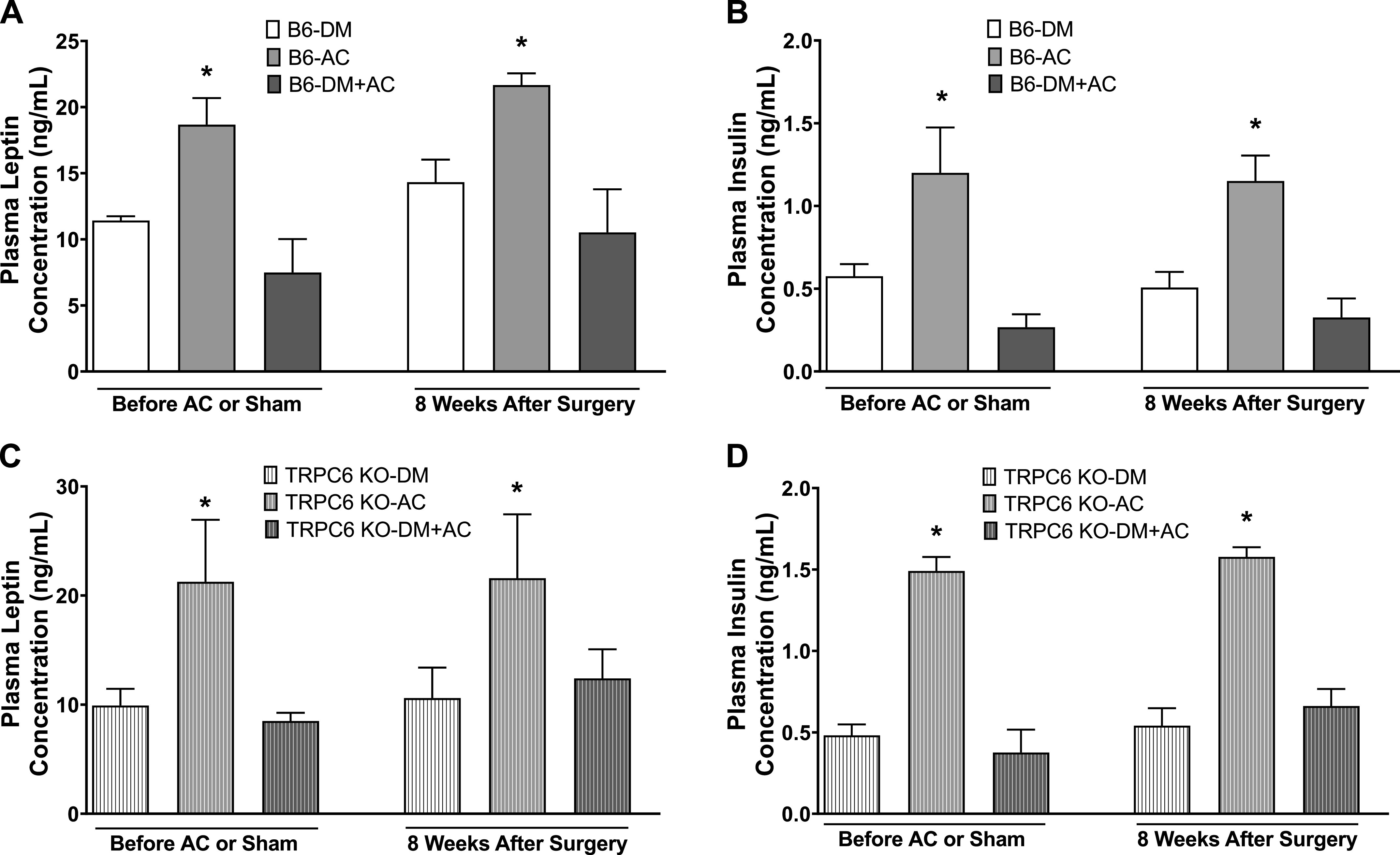
Plasma leptin and insulin concentrations in different groups of male wild-type (WT; B6) and transient receptor potential cation channel 6 (TRPC6) knockout (KO) mice before and after aortic constriction (AC) or sham surgery. *A*: plasma leptin concentrations in diabetes mellitus (DM), AC, and DM + AC groups of WT mice before AC or sham surgery and 8 wk after the surgery. *B*: plasma insulin levels in DM, AC, and DM + AC groups of WT mice before AC or sham surgery and 8 wk after the surgery. *C*: plasma leptin concentrations in DM, AC, and DM + AC groups of TRPC6 KO mice before AC or sham surgery and 8 wk after the surgery. *D*: plasma insulin concentrations in DM, AC, and DM + AC groups of TRPC6 KO mice before AC or sham surgery and 8 wk after the surgery. *n* = 6–8 per group. *P < 0.05 when comparing AC with DM and DM+AC groups by one-way ANOVA and Tukey’s test. B6, C57BL/6J females.

Plasma leptin (Fig. 2, *A* and *C*) and insulin (Fig. 2, *B* and *D*) levels in diabetic WT and TRPC6 KO mice (DM and DM + AC groups) were significantly lower than in nondiabetic mice (AC group) before and after the AC or sham surgery. Overall, mild type 1 DM in WT and TRPC6 KO mice were created with similar hyperglycemia and metabolic phenotypes when measured before and after the AC or sham surgery.

### Impact of AC on BP, HR, and Kidney and Heart Weights

**Figure 3.**
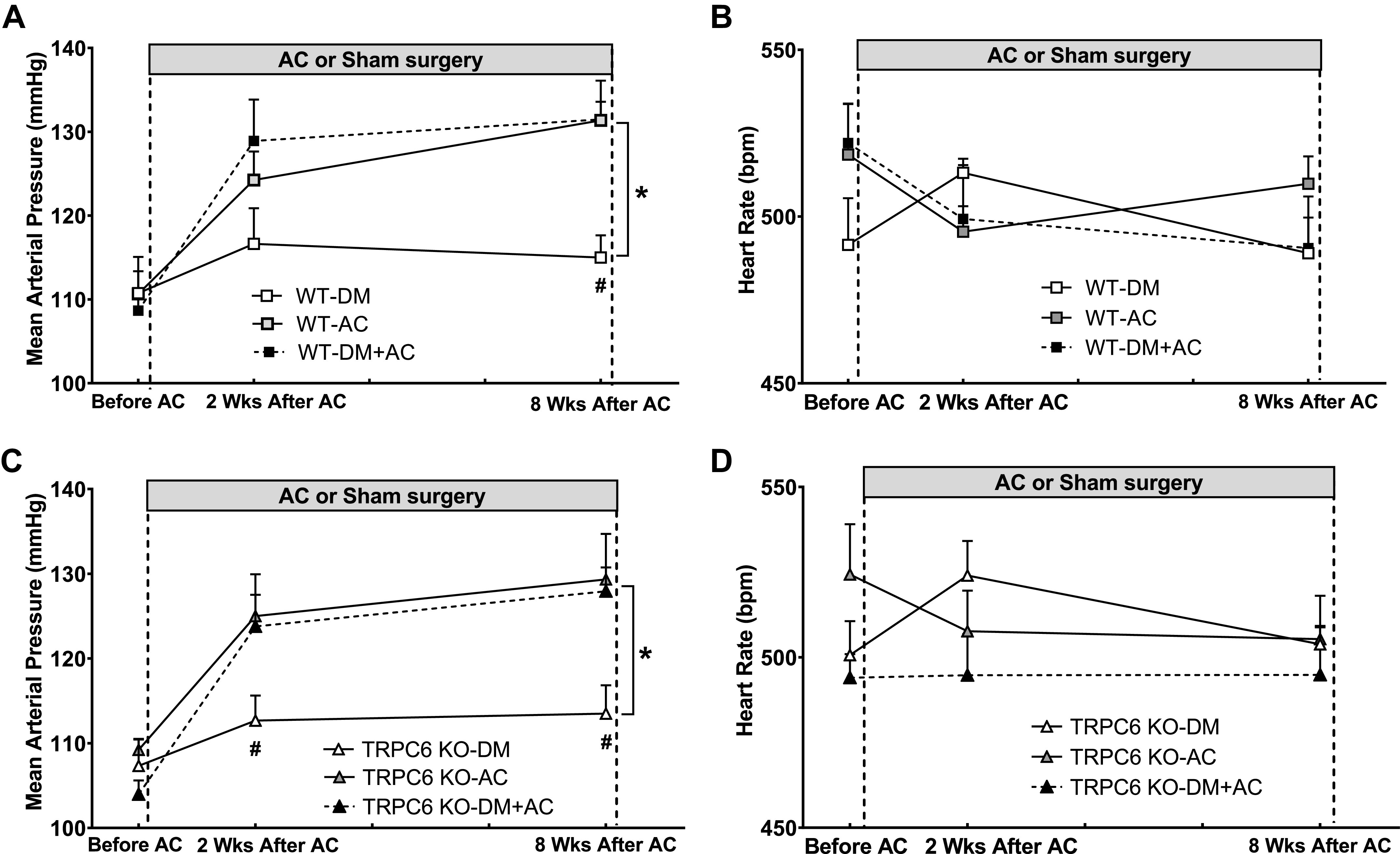
Blood pressure and heart rate [in beats/min (bpm)] in different groups of male wild-type (WT) and transient receptor potential cation channel 6 (TRPC6) knockout (KO) mice before and after aortic constriction (AC) or sham surgery. *A*: mean arterial pressure measured by telemetry in diabetes mellitus (DM), AC, and DM + AC groups of WT mice before AC or sham surgery and at 2 and 8 wk after surgery. *B*: heart rate measured in DM, AC, and DM + AC groups of WT mice before AC or sham surgery and at 2 and 8 wk after surgery. *C*: mean arterial pressure measured by telemetry in DM, AC, and DM + AC groups of TRPC6 KO mice before AC or sham surgery and at 2 and 8 wk after the surgery. *D*: heart rate measured in DM, AC, and DM + AC groups of TRPC6 KO mice before AC or sham surgery and at 2 and 8 wk after surgery. *n* = 6–8 per group. *P < 0.05 when comparing AC vs. DM or DM + AC mice by two-way ANOVA. #*P* < 0.05 when comparing AC vs. DM or DM + AC mice by Tukey’s post hoc test at 1 wk before AC or sham surgery and at 2 and 8 wk after AC or sham surgery.

Baseline BP was similar in all groups of WT (MAP: 109 ± 2 mmHg) and TRPC6 KO mice (MAP: 108 ± 2 mmHg) before the AC or sham surgery. AC significantly increased MAP in all groups of AC and DM + AC mice by an average of ∼25 mmHg. As shown in Fig. 3, *A* and *C*, MAP increased from 106 ± 4 mmHg to 131 ± 3 mmHg at 8 wk after AC in WT-DM + AC mice. In TRPC6 KO-DM + AC mice, AC induced a similar increase in MAP, from 104 ± 2 mmHg to 128 ± 4 mmHg after 8 wk. AC had no significant effect on HR in any of the groups (Fig. 3, *B* and *D*). At the end of the in vivo experiments, we collected and measured the weight of the heart and of the left and right kidneys from each group of WT and TRPC6 KO mice. In both WT and TRPC6 KO mice, AC and DM + AC mice had heavier heart weights than DM mice (Table 3). Weights of the left and right kidneys in each group of WT and TRPC6 KO mice were similar, although the right kidneys in AC and DM + AC groups were heavier than the left kidneys in both WT and TRPC6 mice (Table 2).

**Table 3.** Heart and kidney weights in different groups of male WT and TRPC6 KO mice at 8 wk after AC or sham surgery

	WT-DM	WT-AC	WT-DM + AC	TRPC6 KO-DM	TRPC6 KO-AC	TRPC6 KO-DM + AC
Heart weight, g	0.13 ± 0.01	0.16 ± 0.01†	0.17 ± 0.02†	0.15 ± 0.01	0.19 ± 0.02†	0.18 ± 0.01†
Left kidney weight, g	0.19 ± 0.02	0.18 ± 0.01	0.19 ± 0.01	0.19 ± 0.01	0.16 ± 0.02	0.20 ± 0.01
Right kidney weight, g	0.21 ± 0.02	0.22 ± 0.01*	0.23 ± 0.02*	0.20 ± 0.01	0.20 ± 0.01*	0.23 ± 0.01*

Values are means ± SE; *n* = 6–9. AC, aortic constriction; DM, diabetes mellitus; KO, knockout; TRPC6, transient receptor potential cation channel 6; WT, wild-type. *P < 0.05 when comparing left and right kidney weights in the same group of WT or TRPC6 KO mice by *t* test. †*P* < 0.05 when comparing heart weight in AC and DM + AC with DM mice in WT and TRPC6 KO mice by one-way ANOVA.

### TRPC6 Deficiency Attenuates Increased Urinary Albumin Excretion in Mice Exposed to Both Hyperglycemia and HTN

In WT and TRPC6 KO mice, 24-h total urine output was significantly higher in DM and DM + AC groups of mice than in nondiabetic AC mice (5.0 ± 1.4 mL/day in WT-DM mice and 5.1 ± 1.5 mL/day in WT-DM + AC mice compared with 1.7 ± 0.2 mL/day in WT-AC mice). There were no significant differences in urine output in the DM and DM + AC groups between WT and TRPC6 KO mice before and after the AC or sham surgery.

**Figure 4.**
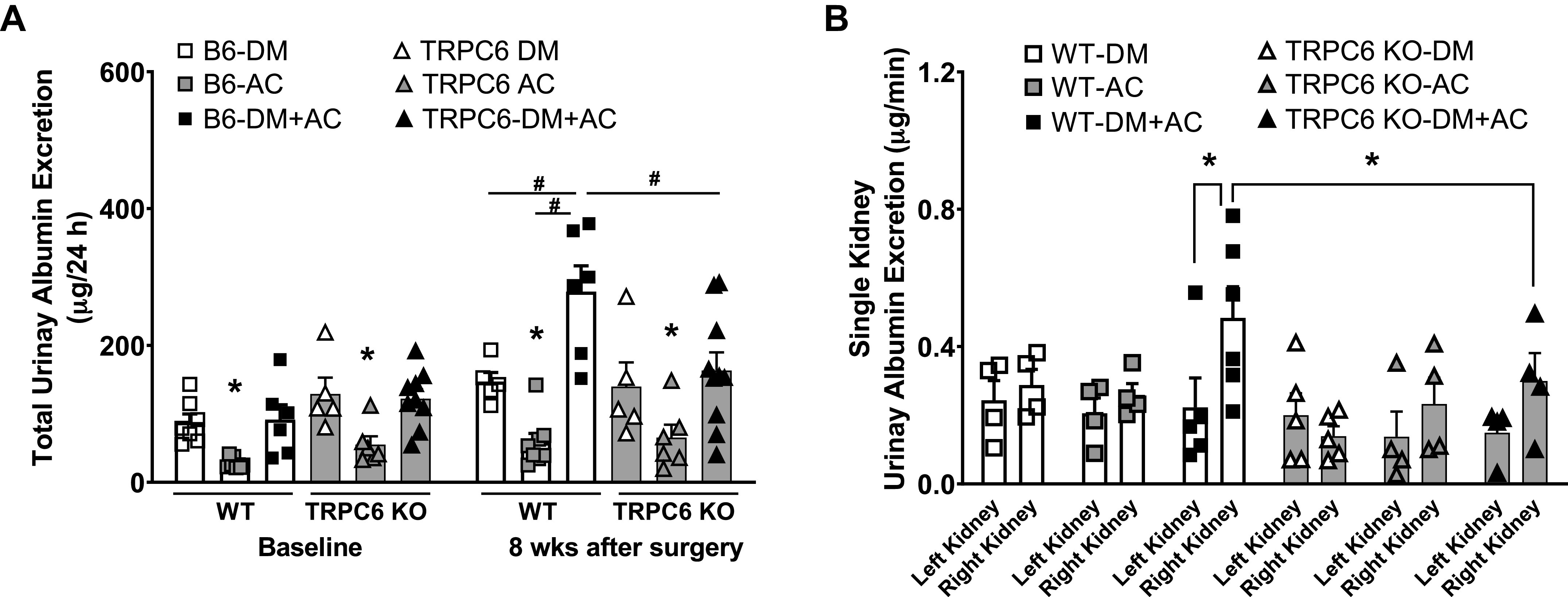
Total 24-h and single kidney urinary albumin excretion in different groups of male wild-type (WT; B6) and transient receptor potential cation channel 6 (TRPC6) knockout (KO) mice before and after 8 wk after aortic constriction (AC) or sham surgery. *A*: 24-h urinary albumin excretion at the baseline and after AC or sham surgery in diabetes mellitus (DM), AC, and DM + AC groups of WT and TRPC6 KO mice. *n* = 5–6 per group. *P < 0.05 when comparing urinary albumin excretion in AC mice vs. DM or DM + AC mice by one-way ANOVA. #*P* < 0.05 when comparing urinary albumin excretion in WT-DM + AC mice vs. WT DM and WT-AC mice in WT and TRPC6 KO-DM + AC mice after AC or sham surgery by one-way ANOVA followed by a Bonferroni’s test. *B*: single left and right kidney urinary albumin excretion in DM, AC, and DM + AC groups of WT and TRPC6 KO mice at 8 wk after AC or sham surgery. *n* = 4 or 5 per group. *P < 0.05 when comparing the left and right kidneys in WT-DM + AC mice and with the right kidney from TRPC6 KO-DM + AC mice by two-way ANOVA followed by a Bonferroni’s test. B6, C57BL/6J females.

Twenty-four-hour urinary albumin excretion results showed that during baseline, before AC, urinary albumin excretions in diabetic mice (WT-DM and WT-DM + AC) were significantly higher than in nondiabetic WT mice (87.9 ± 11.9 µg/24 h in DM mice and 91.7 ± 21.6 µg/24 h in DM + AC mice vs. 27.7 ± 2.7 µg/24 h in AC mice). After 8 wk of sham surgery, urinary albumin excretion in WT DM mice increased further to 147.1 ± 13.4 µg/24 h. In WT-AC mice, urinary albumin excretion was slightly increased to 58.3 ± 13.1 µg/24 h after 8 wk of HTN. However, the combination of HTN and DM caused marked increases in 24-h urinary albumin excretion to 278.8 ± 37.7 µg/24 h in WT-DM + AC mice (Fig. 4*A*). In TRPC6 KO mice, urinary albumin excretion in diabetic TRPC6 KO-DM and DM + AC mice was significantly higher than in nondiabetic TRPC6 KO-AC mice during the baseline and were similar to WT diabetic mice. Importantly, urinary albumin excretion in TRPC6 KO-DM + AC mice after 8 wk of AC was only slightly increased and was significantly lower than in WT-DM + AC mice after 8 wk of AC (163.3 ± 13.1 vs. 278.8 ± 37.7 µg/24 h in TRPC6 KO-DM + AC and WT-DM + AC mice, respectively; Fig. 4*A*).

Measurement of total 24-h urinary albumin from both kidneys, however, does not differentiate which kidney is responsible for the large increases in urinary albumin in WT-DM + AC mice. Therefore, we further measured urinary albumin excretion from the left and right kidneys in DM, AC, and DM + AC groups of WT and TRPC6 KO mice at 8 wk after the AC or sham surgery. As shown in Fig. 4*B*, urinary albumin excretion was markedly increased in the right kidneys of WT-DM + AC mice (0.5 ± 0.1 µg/min, exposed to hyperglycemia and high BP) compared with the left kidneys of the same animals (0.2 ± 0.1 µg/min, exposed only to hyperglycemia) as well as the right kidneys from WT-AC mice (0.3 ± 0.1 µg/min, exposed only to high BP) and the right kidneys in WT-DM mice (0.3 ± 0.1 µg/min, exposed only to hyperglycemia). In contrast, urinary albumin excretion in the right kidneys of TRPC6 KO-DM + AC mice was significantly less than in the right kidneys of WT-DM + AC mice (0.3 ± 0.1 vs. 0.5 ± 0.1 µg/min). These results indicate that increased urinary albumin in DM + AC mice mainly occurs in the right kidneys exposed to both increased BP and hyperglycemia. Deletion of TRPC6 attenuates renal albumin excretion induced by the combination of HTN and DM.

### TRPC6 Deficiency Attenuates the Decline in GFR in the Kidneys of Mice Exposed to Both Hyperglycemia and HTN

**Figure 5.**
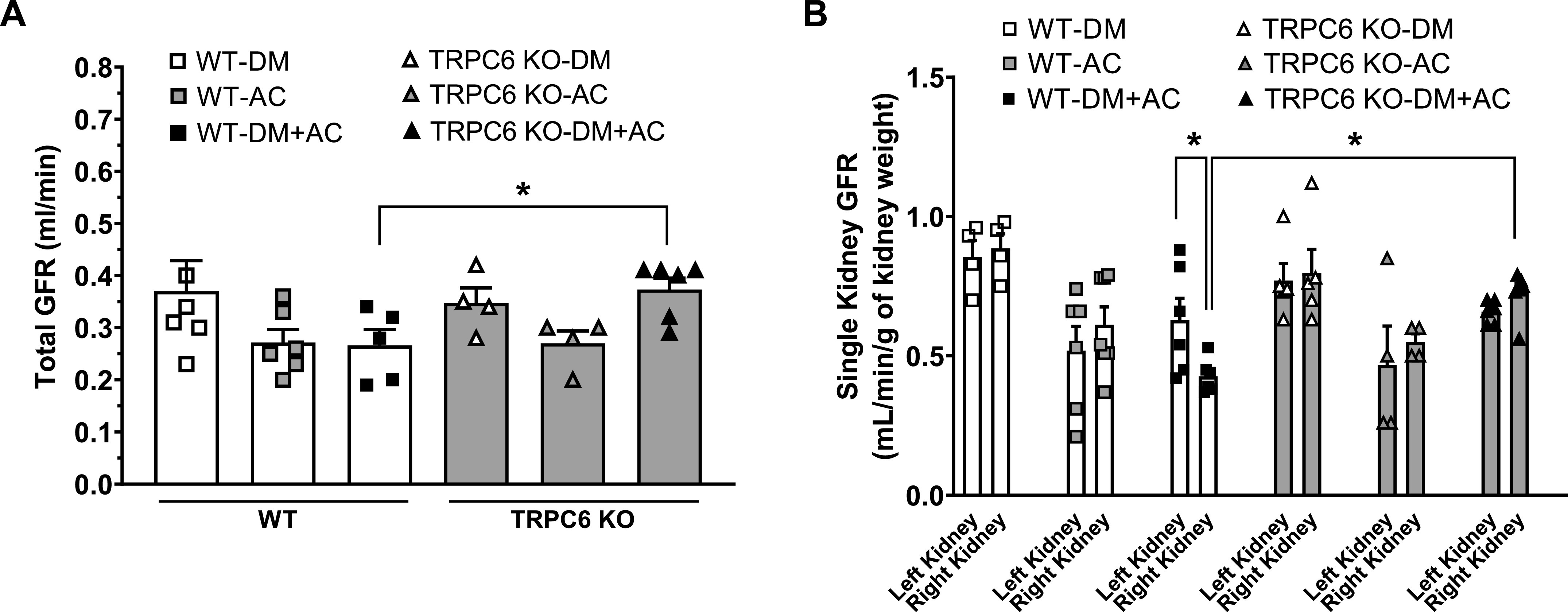
Total and single kidney glomerular filtration rate (GFR) in different groups of male wild-type (WT) and transient receptor potential cation channel 6 (TRPC6) knockout (KO) mice at 8 wk after aortic constriction (AC) or sham surgery. *A*: total GFR before and after AC or sham surgery in diabetes mellitus (DM), AC, and DM + AC groups of WT and TRPC6 KO mice. *n* = 4–6 in each group. *P < 0.05 when comparing GFR between WT-DM + AC and TRPC6 KO-DM + AC mice by *t* test. *B*: single left and right kidney GFR in DM, AC, and DM + AC groups of WT and TRPC6 KO mice at 8 wk after AC or sham surgery. *n* = 4–6 per group. *P < 0.05 when comparing the left and right kidneys in WT-DM + AC mice and with the right kidneys from TRPC6 KO-DM + AC mice by two-way ANOVA followed by a Bonferroni’s test.

To evaluate the interactions of HTN and hyperglycemia on kidney function, total GFR and single left and right GFR were measured in DM, AC, and DM + AC groups of WT and TRPC6 KO mice at 8 wk after the AC or sham surgery. Our results show that total GFR in WT-DM + AC mice tended to be lower than in WT DM mice. When we compared WT and TRPC6 KO mice, total GFR in WT mice with DM + AC was also significantly reduced compared with TRPC6 KO mice with DM + AC (0.27 ± 0.03 vs. 0.37 ± 0.02 mL/min; Fig. 5*A*).

Left and right single-GFR measurements showed that after 8 wk of AC, average GFR in the right kidneys of WT DM + AC mice was significantly lower than in the left kidneys of the same animals. GFR in the right kidney of WT DM + AC mice was also significantly lower than GFR in the right kidneys of WT-DM mice exposed to only high glucose. This result further suggests that increased BP and hyperglycemia together may promote a rapid decline of GFR. In contrast, GFR in the right kidneys of TRPC6 KO DM + AC mice was preserved, despite being exposed to HTN and hyperglycemia, and had similar values as in the left kidneys of the same DM + AC mice and in the right kidneys of DM mice. Importantly, average GFR in the right kidneys of TRPC6 KO DM + AC mice was significantly higher than in the right kidneys of WT-DM + AC mice (0.7 ± 0.1 vs. 0.4 ± 0.1 mL/min/g kidney wt; Fig. 5*B*), suggesting that TRPC6 deficiency may markedly attenuate the decline of GFR in kidneys exposed to both hyperglycemia and high BP.

### TRPC6 Deficiency Attenuates Renal Glomerular Structural Changes Induced by the Combination of Hyperglycemia and HTN

**Figure 6.**
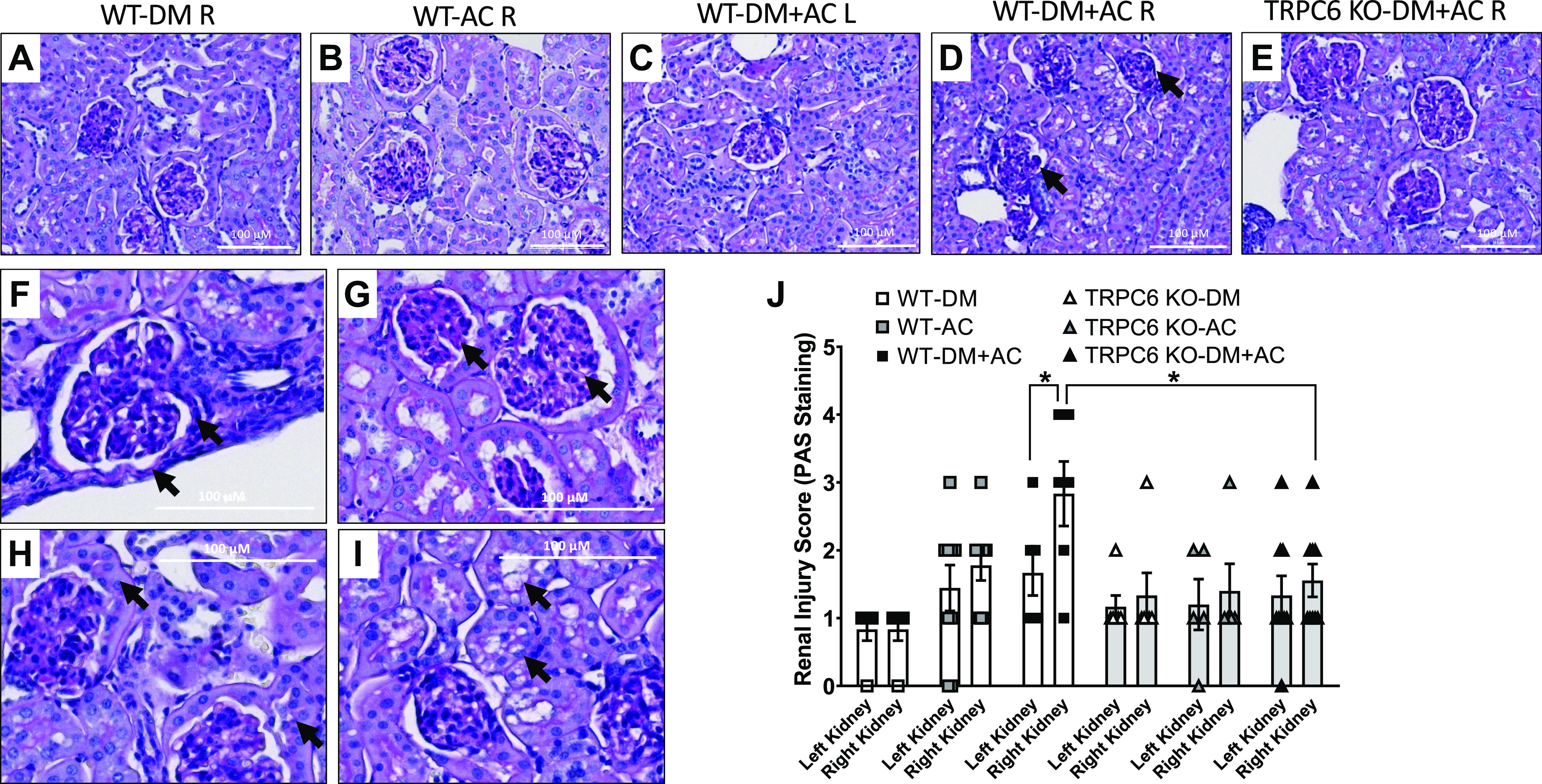
Renal morphological changes in different groups of male wild-type (WT) and transient receptor potential cation channel 6 (TRPC6) knockout (KO) mice at 8 wk after aortic constriction (AC) or sham surgery. Representative images of periodic acid-Schiff (PAS) staining of kidney slices from the right kidney of WT diabetes mellitus (DM) mice (*A*), right kidney of WT-AC mice (*B*), left kidney of WT-DM + AC mice (*C*), right kidney of WT-DM + AC mice (*D*), and right kidney of TRPC6 DM + AC mice (*E*). Glomerular and tubular injury can be observed in diabetic-hypertensive right kidneys in WT-DM + AC mice as indicated by arrows. *F−I*: representative images showing glomerular and tubular injury in the right kidneys of WT-DM + AC mice in magnified views. *F*: increased thickness of Bowman’s capsule. *G*: mesangial expansion (left glomerular) and capillary wall thickening (right glomerular). *H*: cellular proliferation of parietal cell in Bowman’s capsule. *I*: tubular cell injury. *J*: renal injury was scored based on the renal morphological changes at 8 wk of AC or sham surgery in DM, AC, and DM + AC groups of WT and TRPC6 KO mice. *n* = 5–7 per group. *P < 0.05 when comparing the left and right kidney in WT-DM + AC mice and with the right kidney from TRPC6 KO-DM + AC mice by a Kruskal-Wallis nonparametric test followed by a Dunn’s multiple-comparisons test.

As shown in Fig. 6, *A–E*, the right kidneys from WT-DM + AC mice, which were exposed to both hyperglycemia and high BP, had more severe renal injury compared with the left kidneys from the same animals. Damage in the right kidneys of WT-DM + AC mice was also greater than in the right kidneys from diabetic WT-DM mice and from hypertensive WT-AC mice. Marked renal morphological changes, such as thickening of Bowman’s capsule, increased glomerular mesangial matrix and cellularity, glomerular capillary wall thickening, metaplasia of epithelial cells in Bowman’s capsule, and tubular cell injury, were observed in the right kidneys of WT-DM + AC mice (Fig. 6, *F–I*). We also compared renal injury scores in the right kidneys of TRPC6 KO-DM + AC mice and WT-DM + AC mice. Even though the kidneys of TRPC6 KO mice were exposed to similar hyperglycemia and high BP, they had much less injury than observed in WT mice. As shown in Fig. 6*J*, the overall kidney injury score in the right kidneys of WT-DM + AC mice was significantly higher than in the left kidneys of WT-DM + AC mice and in the right kidneys of TRPC6 KO-DM + AC mice.

### TRPC6 Deficiency Attenuates Renal Apoptotic Injury Induced by the Interaction of Hyperglycemia and HTN

**Figure 7.**
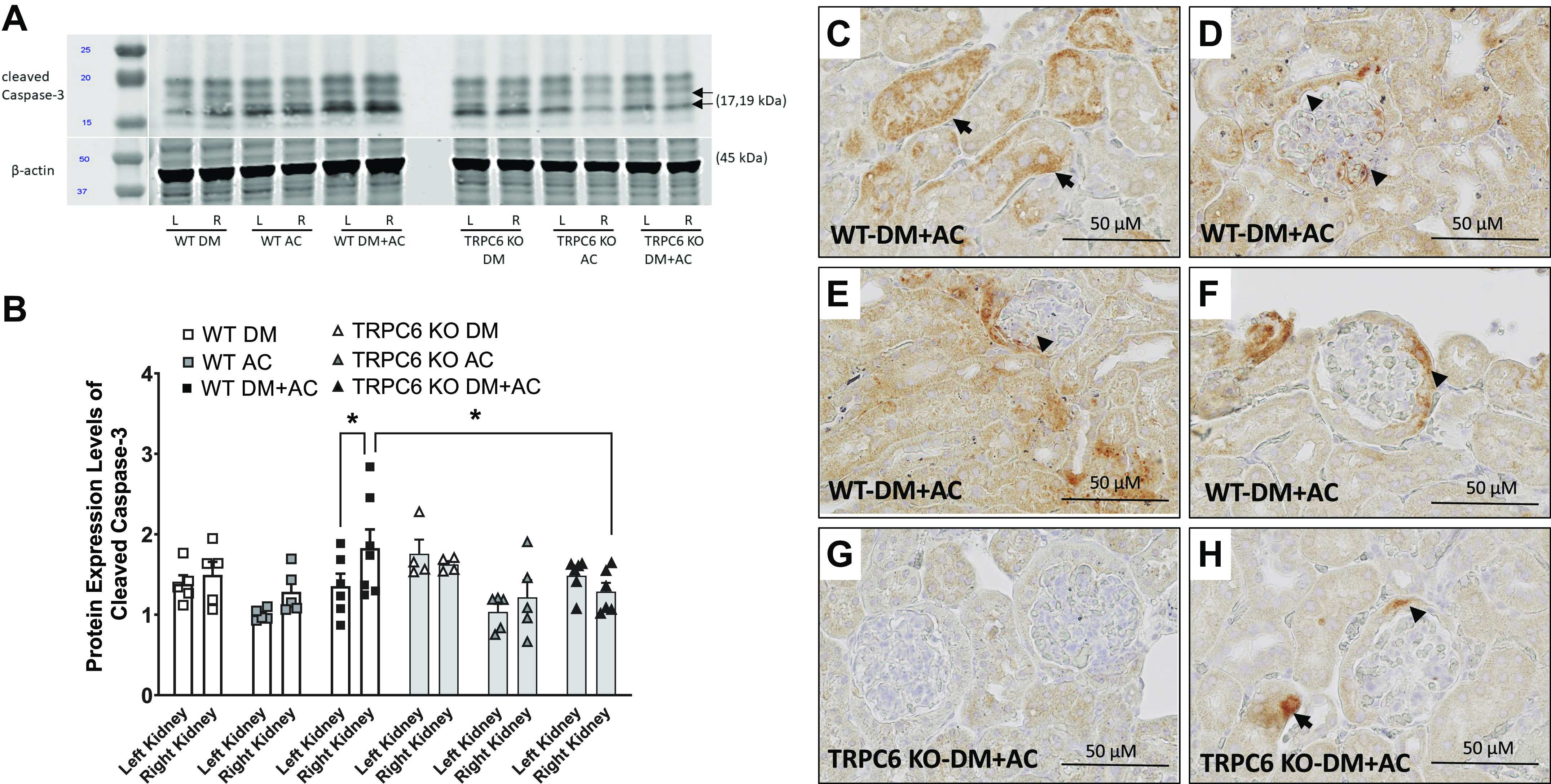
Measurement of apoptosis in different groups of male wild-type (WT) and transient receptor potential cation channel 6 (TRPC6) knockout (KO) mice at 8 wk after aortic constriction (AC) or sham surgery. *A*: immunoblot examples of renal cortex cleaved caspase-3 expression in diabetes mellitus (DM), AC, and DM + AC groups of WT and TRPC6 KO mice. *B*: optical density analysis of cleaved caspase-3 expression in DM, AC, and DM + AC groups of WT and TRPC6 KO mice after 8 wk of AC or sham surgery. *n* = 4 or 5 per group. *P < 0.05 when comparing the left and right kidney in WT-DM + AC mice and with the right kidney from TRPC6 KO-DM + AC mice by two-way ANOVA followed by a Bonferroni’s test. Representative images show the positive staining for cleaved caspase-3 from the right kidneys of WT-DM + AC mice (*C−F*) and TRPC6 KO-DM + AC mice (*G* and *H*) after 8 wk of AC surgery. Arrows indicate apoptotic cells in renal tubules; arrowheads indicate apoptotic cells in the glomerulus.

To investigate the potential molecular pathways that mediate the effects of HTN and hyperglycemia on kidney injury, an apoptosis marker, cleaved caspase-3 protein, was measured by Western blot analysis in kidney cortex homogenates of DM, AC, and DM + AC groups of WT and TRPC6 KO mice. Cleaved caspase-3 expression in the right kidneys of WT-DM + AC mice exposed to high BP and hyperglycemia was significantly higher than in kidneys from other groups of mice (Fig. 7, *A* and *B*). Importantly, cleaved caspase-3 expression in the right kidneys of TRPC6 KO-DM + AC mice was significantly lower than in the right kidneys of WT-DM + AC mice, suggesting that TRPC6 deficiency may protect the kidney from diabetic-hypertensive injury with reduced apoptosis. To determine the location of apoptotic cells in DM + AC mice, we performed immunohistochemistry staining for cleaved caspase-3 in the right kidneys of WT-DM + AC mice (Fig. 7, *C–F*) and TRPC6 KO-DM + AC mice (Fig. 7, *G* and *H*). Stronger cleaved caspase-3 staining was observed in the glomerulus, epithelial cell layer of Bowman’s capsule, and renal tubular cells in the right kidneys of WT-DM + AC mice than in TRPC6 KO mice. These results suggest that the combination of hyperglycemia and high BP induces apoptotic cell injury in glomerular and renal tubules.

## DISCUSSION

A major new finding of our study is that the combination of moderate DM and HTN promoted marked renal dysfunction, albuminuria, and apoptotic cell injury and that these effects were greatly ameliorated by TRPC6 deficiency. Although moderate DM in the absence of HTN caused minimal renal injury, substantial kidney injury was apparent in only 8 wk when DM coexisted with moderate increases in BP in WT mice. In mice with TRPC6 deficiency, the renal dysfunction, albuminuria, glomerular injury, and apoptosis in kidneys exposed to both HTN and hyperglycemia were greatly attenuated. These findings suggest that TRPC6 may have an important role in mediating kidney injury in diabetic-hypertensive kidney disease.

### Mouse Model With DM and Kidneys Exposed to Normal or Elevated BP in the Same Animal

We previously reported that moderate HTN promoted significant injury in kidneys of type 2 diabetic Goto-Kakizaki animals, whereas little injury was observed when the kidneys were protected from elevated BP. In addition, moderate HTN for 8 wk caused minimal kidney damage in the absence of DM (17). Thus, DM and increased BP appear to interact to induce kidney injury, whereas only modest kidney injury occurs when DM occurs in the absence of HTN.

To further understand the mechanisms by which HTN interacts with DM to promote kidney injury and to avoid complicating effects of obesity and hyperinsulinemia associated with most type 2 DM models, we used a type 1 DM model in this study. We induced a moderate, stable form of type 1 DM by intravenous injection of a moderate dose of STZ than is often used to produce DM in mice (18). We induced HTN by constriction of the aorta between the renal arteries so that the right kidney was exposed to increased BP, whereas the left kidney had normal or slightly reduced BP. Therefore, this model permits direct assessment of the chronic effects of DM in kidneys exposed to normal or increased BP in the same animal; both kidneys are exposed to the same level of glucose, circulating hormones, and other blood-borne and neural influences to different levels of perfusion pressure.

Our results indicate that maintenance of blood glucose concentrations of 250–350 mg/dL for 15 wk did not cause major kidney dysfunction or injury in WT or TRPC6-deficient mice as assessed by measurements of GFR, albuminuria, apoptosis markers, and various histological assessments. This result matches a previous study showing no protective effect of TRPC6 inactivation on albuminuria in STZ-induced diabetic Sprague-Dawley rats (19). It is possible that the increased albuminuria in diabetes is not TRPC6 related, although some studies have indicated that diabetes alone, in the absence of HTN, does not cause marked increases in kidney injury, similar to our results (17, 20). These findings are also consistent with an observational study in humans indicating that in the early stages of diabetic nephropathy, before the development of marked increases in BP, there is glomerular hyperfiltration and albuminuria but mild renal injury (21).

### Interaction of DM and HTN to Promote Renal Injury

Previous studies have suggested that the superimposition of HTN on type 1 or type 2 DM produces much more severe kidney injury than DM alone (17, 20). Each 10 mmHg increase in mean systolic BP was associated with a 15% increase in the hazard ratio for developing micro- and macroalbuminuria and impaired kidney function (6). In fact, increased BP may be a prerequisite for the rapid progression of diabetic nephropathy. Researchers have tried to replicate the hemodynamic effects of DM and HTN in rodent models by administering high-protein diets or by uninephrectomy, as both elevate glomerular hydrostatic pressure, or by superimposing DM on genetic models of HTN (22). For example, in transgenic rodents with an excessively activated renin-angiotensin-aldosterone system to induce HTN (23), there is an accelerated development of diabetic nephropathy. However, it has been challenging in these studies to separate potential contributions of neural, hormonal, metabolic, and other factors from BP effects on the kidneys. Furthermore, the cellular and molecular mechanisms by which hemodynamic effects may amplify hyperglycemic effects in causing kidney injury are still poorly understood.

We found in the present study that 8 wk of HTN, in the absence of DM, caused only minimal albuminuria and renal injury in WT or TRPC6 KO mice in the absence of DM. However, the coexistence of HTN and hyperglycemia exerted either synergistic effects to promote renal dysfunction and injury as reflected by increases in 24-h urinary albumin excretion, reduced GFR, or additive effects to exacerbate histological injury in glomeruli and tubules in the right kidneys of WT mice with DM and exposed to increased BP. In contrast, the left kidneys of WT-DM + AC mice, which were exposed to hyperglycemia and normal BP, had only mild renal dysfunction and injury similar to the kidneys in control WT-DM mice. This result is consistent with a previous study showing in Long–Evans rats that the combination of two kidneys-one clip-induced HTN and STZ-induced type 1 DM caused minor injury in the clipped kidney and severe glomerular and vascular injury in the nonclipped kidney (20).

### Hemodynamic Factors Could Contribute to Kidney Injury Caused by DM + HTN

There is compelling evidence that hyperglycemia impairs normal autoregulation of GFR and renal blood flow (24, 25). To the extent that renal autoregulation is impaired, increases in systemic arterial pressure would be transmitted to the glomerular capillaries in diabetic kidneys to a greater degree than in normal kidneys and could lead to kidney injury. GFR was significantly reduced and urinary albumin excretion was increased in the right kidneys (exposed to elevated BP) of WT-DM + AC mice after 8 wk of HTN. This rapid decrease of GFR in the hypertensive kidneys of WT-DM + AC mice is similar to the decline observed in patients with diabetes, who first undergo glomerular hyperfiltration followed by reductions of GFR that are associated with nephron injury preceding a further decline of GFR to subnormal levels as diabetic nephropathy progresses (26). In contrast, the left kidneys (protected from elevated BP) of WT-DM + AC mice had minimal injury despite being exposed to DM for 15 wk, a significant part of a mouse’s lifespan. Therefore, one potential mechanism for the synergy of HTN and DM to promote kidney injury may be the greater mechanical stress on the glomerular capillaries due to impaired renal autoregulation, renal vasodilation, glomerular hyperfiltration, and greater transmission of increases in systemic arterial pressure to the glomerulus.

### Potential Role of TRPC6 in Mediating Kidney Injury When DM and HTN Occur Together

The high pressure of glomerular capillaries maintains outward flow of the plasma fluid while causing tensile stress on the capillary endothelium and podocytes. In parallel, continuous flow of the ultrafiltrate into Bowman’s space generates shear stress on podocytes and epithelial cells (11). Thus, when increased BP adds to DM-induced hyperfiltration, long-lasting intraglomerular mechanical challenges, including glomerular HTN, hyperfiltration, hypertrophy, and outflow of glomerular filtrate from subpodocyte spaces, may promote progressive podocyte loss through detachment from the glomerular basement membrane and ultimately complete loss of podocytes in advanced chronic kidney disease with overt proteinuria (27–29).

Transduction of mechanical forces into biochemical signals requires “mechanosensor” proteins. These proteins sense mechanical forces and induce biochemical changes of the mechanosensory complex, including ion channels and anchor proteins that provide cell-cell or cell-matrix linkage (30, 31). TRPC6 is highly selective for Ca^2+^ over other cations and has been implicated in Ca^2+^-dependent processes in the peripheral vasculature, kidney, and heart (32). There is evidence that TRPC6 may interact with nephrin, podocin, and mechanosensitive Ca^2+^-activated K^+^ channels (large-conductance Ca^2+^-activated K^+^ channels) in the kidney, allowing Ca^2+^ influx to modulate nephrin signaling and cytoskeletal dynamics (33, 34). However, whether TRPC6 channels are directly or indirectly gated by mechanical factors is still controversial (35). TRPC6 can be activated in response to G protein-mediated signaling cascades associated with phospholipase C activation (36, 37). TRPC6 activation may also be enhanced by the combination of receptor binding and mechanical stimulation and has been reported to play an important role in mediating kidney injury in DM (38). Another study has suggested that activation of TRPC6 channels is linked to renal injury and may contribute to the development of chronic kidney disease in STZ-induced type 1 diabetic Dahl salt-sensitive rats (39). Whether TRPC6 activation mediates renal injury induced by DM and HTN, however, is still unclear (40). Our data also suggest that TRPC6 may contribute to the development of renal injury associated with activation of the apoptosis pathway when hyperglycemia and high BP coexist. The cellular mechanisms that lead to apoptosis in kidneys subjected to hyperglycemia and HTN are unclear but may be related, in part, to increased reactive oxygen species (ROS) production. Previous studies have suggested that the NADPH oxidase NOX4 produces ROS that could activate TRPC6-mediated Ca^2+^ influx in podocytes and that podocytes from TRPC6 KO mice have attenuated glomerular injury response to H_2_O_2_-induced Ca^2+^ influx (41).

The role of Ca^2+^ signals in apoptosis has been studied intensively and further reinforced by the demonstration that antiapoptotic proteins, such as BCL-2, lower endoplasmic reticulum Ca^2+^ levels and reduce cytosolic and mitochondrial Ca^2+^ responses to extracellular stimuli by increasing endoplasmic reticulum Ca^2+^ leak (42). However, the detailed molecular mechanisms that mediate Ca^2+^ influx through TRPC6 activation in DM and HTN and that cause the release of caspase cofactors from the organelle, resulting in apoptotic cell death, are still unclear and warrant further study. Although our study was not designed to assess the role of TRPC6 in podocytes to promote glomerular injury, the finding that TRPC6 KO reduced apoptotic injury in both glomerular and renal tubular cells suggests the TRPC6 may contribute to injury in different kidney cell types when DM is combined with HTN. A recent study has shown that high glucose reduces podocin protein levels by activating TRPC6 and induces morphological changes of cultured podocytes (43). TRPC6 may also enhance activation of calpain, a Ca^2+^-dependent protease, that contributes to podocyte injury using an in vitro podocyte model (44, 45). Therefore, further studies are needed to determine the specific roles of TRPC6 in glomerular capillary endothelial cells, podocytes, and tubular cells in promoting kidney injury.

TRPC6 expression is found in several cell types in the kidney, including podocytes, mesangial cells, renal tubular cells, and possibly renovascular smooth muscle cells (7). TRPC6 is also expressed in a variety of other cell types, such as neurons (46) and immune cells, including macrophages, T cells, and neutrophils (47–49). One limitation of our study is that global KO of TRPC6 may impact several cell types that normally express TRPC6, which may contribute to BP regulation and the development of kidney injury. Dietrich et al. (11) reported increased vascular smooth muscle contractility in TRPC6 KO with increased MAP compared with WT mice. In contrast, Ding et al. (50) showed that H_2_O_2_ stimulated a dose-dependent constriction of the aortas from WT mice but not from the vessels of TRPC6 KO mice. In addition, Eckel et al. (51) showed that TRPC6-deficient mice had normal BP and albumin excretion rates, which matches with our present study. These apparently conflicting results may be due to differences in age or methods used to quantify these variables. These observations may also reflect a more complex role for TRPC6 in different organs or cell populations. Our study showed that TRPC6 contributes to kidney injury induced by DM and HTN; however, the detailed molecular mechanisms are still unclear, and future studies using tissue-specific TRPC6 KO mice are warranted.

### Perspectives

Our results indicate that even mild HTN and DM can interact to promote renal dysfunction, albuminuria, glomerular sclerosis, and apoptotic cell injury, making it a highly translational model to human patients with diabetic nephropathy. Deletion of TRPC6 markedly attenuated renal dysfunction and reduced apoptotic cell injury in glomeruli and tubules of kidneys exposed to hyperglycemia and high BP. However, the molecular mechanisms by which HTN-induced mechanical forces interact with high glucose to promote apoptosis and renal injury are still not fully understood and warrant further investigation. Furthermore, the roles of TRPC6-mediated Ca^2+^ influx in contributing to injury of different renal cell populations when DM and HTN coexist are unclear. Investigating the impact of TRPC6 deletion in specific renal cells and the molecular pathways affected will provide additional insights into the pathogenesis of kidney injury in DM and HTN. However, an important implication of our study is that simultaneous tight control of HTN and hyperglycemia may be required to slow or prevent the progression of diabetic nephropathy. Inhibition of TRPC6 activation may also be a potential therapeutic strategy for diabetic nephropathy.

## GRANTS

This work was supported by National Institutes of Health Grants P01HL51971, R00DK113280, R01DK121411, P20GM104357, and U54GM115428.

## DISCLOSURES

No conflicts of interest, financial or otherwise, are declared by the authors.

## AUTHOR CONTRIBUTIONS

Z.W., Y.F., J.M.d.C., and J.E.H. conceived and designed research; Z.W., Y.F., and J.S. performed experiments; Z.W., Y.F., and J.S. analyzed data; Z.W. and Y.F. interpreted results of experiments; Z.W. and J.S. prepared figures; Z.W. drafted manuscript; Z.W., Y.F., J.M.d.C., A.A.d.S., X.L., A.M., A.C.M.O., J.S., and J.E.H. edited and revised manuscript; Z.W., Y.F., J.M.d.C., A.A.d.S., X.L., A.M., A.C.M.O., J.S., and J.E.H. approved final version of manuscript.
